# The Most Common Side Effects Experienced by Patients Were Receiving First Cycle of Chemotherapy

**Published:** 2018-08

**Authors:** İnsaf ALTUN, Alper SONKAYA

**Affiliations:** 1. Dept. of Fundamentals in Nursing, Faculty of Health Sciences, İstinye University, Cevizlibağ, İstanbul, Turkey; 2. Oncology Department, Umraniye Training and Research Hospital, İstanbul, Turkey

## Dear Editor-in-Chief

The side effects of chemotherapy among cancer patients can common and become life-threatening and often occur when patients are at home (1, 2). The side-effects of chemotherapy are a weakening and often unobserved clinical obstacle in cancer management. They can influence the continuance of treatment and, have a negative impact on a patient’s quality of life. Therefore, oncology health professionals need to recognize side effects experienced by their patients and, when possible, help resolve these problems (3).

The current study was conducted to survey the most common side effects experienced by patients were receiving first cycle of chemotherapy. We performed a descriptive study in the University Research and Practice Hospital in Kocaeli, Turkey, during 2016. The study was conducted using a convenience sample of persons followed in the Oncology Outpatient Clinic. Participants were first-time chemotherapy patients with any type and stage of cancer.

Participants volunteered to take in the study and gave an oral informed consent before the study.

A questionnaire prepared by the researchers through review of literature were handed to the patients before chemotherapy regimen. They were asked to complete on the presence of the problems stated in the follow-up form at home. As they did not last long, they did not cause any problem for the patients. The questionnaire for patient potential side effects after chemotherapy was given to patients after chemotherapy regimen. They were asked to complete them on day of the days after the procedure. Statistical analysis was performed using the SPSS, version 16.0 (Chicago, IL, USA).

The study included total of 87 subjects with multiple carcinomas. Mean age of the study participants was 58.8 ± 12.6 yr (range: 30–84 yr), 41.4% (n = 36) were female. With regards to educational status, 60.9% (n = 53) were primary school graduates. 29.9% had breast cancers, 20.7% of them had digestive tract, and 9.2% had urological tract, 8.0% of patients had gynecological tract cancers and 26.4% of them were with cancers other and all patients were receiving first cycle of chemotherapy.

Nausea and vomiting and fatigue was the most frequent side effect experienced (4,5). According to this study the most common reported side effects were nausea and vomiting (%79.3), and fatigue 74.7%. Other frequently reported prominent side effects to include decreased appetite 65.5%, changes in taste 60.9%, hair loss 60.0%, dry mouth 51.7% and constipation 51.7%. Each of these side effects was experienced by more than 50% of the patients. The results obtained from this study are consistent with those of other studies (1,3). Other prominent side effects include diarrhea, numbness or tingling in hands and/or feet, skin changes (e.g. dry skin, redness, itch), fever, damage to the mucosa of the mouth, flu-like symptoms, allergic reaction, memory problems, decreased kidney function, hearing loss and/or ringing in the ears.

The most common chemotherapy-induced side effects are nausea and vomiting, fatigue, decreased appetite, changes in taste, hair loss, dry mouth, and constipation. Management of side effects can be improved the efficacy of tumor therapy. Identification of new treatments to relieve chemotherapy-induced side effects is necessary to develop quality of life amongst cancer patients.
